# Reduced enhancement of high‐frequency component in the cross spectrum of ECG and nostril airflow signals in patients with chronic obstructive pulmonary disease

**DOI:** 10.14814/phy2.12763

**Published:** 2016-04-06

**Authors:** Wan‐An Lu, Jane Kuo, Yan‐Min Wang, Te‐Cheng Lien, Yen‐Bin Liu, Jang‐Zern Tsai, Cheng‐Deng Kuo

**Affiliations:** ^1^ Laboratory of BiophysicsDepartment of Medical ResearchTaipei Veterans General HospitalTaipeiTaiwan; ^2^ Institute of Cultural Asset and ReinventionFo‐Guang UniversityYilanTaiwan; ^3^ Division of Respiratory TherapyDepartment of Chest MedicineTaipei Veterans General HospitalTaipeiTaiwan; ^4^ Medical DepartmentNational Taiwan University HospitalTaipeiTaiwan; ^5^ Department of Electrical EngineeringNational Central UniversityJung‐Li CityTaiwan

**Keywords:** Body mass index, chronic obstructive pulmonary disease, cross‐spectral analysis, electrocardiogram, heart rate variability, respiration

## Abstract

Chronic obstructive pulmonary disease (COPD) is a chronic airway disease with increased airway resistance. This study investigated the common characteristics of electrocardiographic (ECG) and nostril airflow signals in COPD patients using cross‐spectral analysis. Heart rate variability (HRV) measures and cross‐spectral (cs) measures of ECG and nostril airflow were compared in COPD patients and normal subjects, and correlated with their clinical characteristics. We found that cross‐spectral analysis can lead to a significant increase in normalized high‐frequency power (nHFPcs) and a significant decrease in normalized very low‐frequency power (nVLFPcs), normalized low‐frequency power (nLFPcs), and low‐/high‐frequency power ratio (LHRcs) in both normal subjects and COPD patients, as compared with their corresponding HRV measures. Further analysis showed that the percentage increase in nHFP (%nHFP) and the percentage decrease in LHR (%LHR) due to cross‐spectral analysis in COPD patients were significantly smaller than those of normal subjects. All cross‐spectral measures of ECG and nostril airflow in COPD patients did not significantly correlate with their pulmonary function characteristics. However, the nHFPcs correlated significantly and negatively with body mass index (BMI) in both normal subjects and COPD patients, and the %nHFP correlated significantly and negatively with BMI in COPD patients. We conclude that cross‐spectral analysis of ECG and nostril airflow signals could lead to reduced enhancement in the high‐frequency component in the cross spectrum of COPD patients. The magnitude of reduced enhancement in the high‐frequency component in the cross‐spectrum was related to the BMI of the patients. Cross‐spectral analysis of ECG and nostril airflow might be used to assess the cardiovascular‐related functions of COPD patients.

## Introduction

Chronic obstructive pulmonary disease (COPD) is a chronic airway disease whose symptoms and signs include dyspnea and labored breathing. It has been shown that COPD is associated with an increase in vagal activity which can, in turn, explain in part, the reduction in forced expiratory volume in the first second (FEV_1_) and the increase in bronchoconstriction in COPD patients (Volterrani et al. 1994). It has also been shown that chronic hypoxemia can lead to an enhanced cardiac vagal activity and a depressed sympathetic activity in COPD patients (Chen et al. 2006).

Heart rate variability (HRV) offers a noninvasive way to monitor and assess the autonomic nervous control of heart beating over a short period of time, and has been used to predict the morbidity and mortality, to diagnose illness, and to detect autonomic dysfunction in a variety of clinical conditions (Akselrod et al. 1981; Task Force of the European Society of Cardiology, the North American Society of Pacing and Electrophysiology, 1996; Kleiger et al. 2005). HRV is also known as respiratory sinus arrhythmia because the heart rate oscillation can be influenced by the pattern and amplitude of breathing. The respiratory frequency is the frequency at which the high‐frequency peak occurs in the power spectrum of RR intervals (RRI). Thus, examining the association between electrocardiographic (ECG) and respiratory signals may reveal the common characteristics of cardiorespiratory interaction in patients with various kinds of diseases, including COPD.

Power spectral analysis is often used to analyze the power or variance of a signal at different frequencies (Kay and Marple 1981). Cross‐spectral analysis is the extension of single spectral (Fourier) analysis to the simultaneous Fourier spectral analysis of two time series (Rangayyan 2002). Cross‐spectral analysis can uncover the correlations between two time series at different frequencies as a function of frequency, and disclose the information hidden inside those two time series (Rangayyan 2002).

Theoretically, a complete HRV analysis should include both respiratory signals and ECG signals at the same time because respiration is an important ingredient of HRV (Hirsch and Bishop 1981; Brown et al. 1993). We hypothesized that cross‐spectral measures of respiratory and ECG signals might have some clinical implications in COPD patients. The aim of this study was to explore the clinical significance of the cross‐spectral measures of ECG and nostril airflow signals in COPD patients.

## Methods

### Study subjects

Clinically stable and ambulatory COPD patients from the outpatient department of the Taipei Veterans General Hospital were recruited as the study subjects. COPD was defined according to the criteria of the American Thoracic Society (American Thoracic Society, 1987). Age‐matched healthy subjects without cardiopulmonary disease and without using any medication were recruited from the community as the control group.The Institutional Review Board of the Taipei Veterans General Hospital has approved this study, and the written informed consent was obtained from each subject before the study. All subjects were requested not to drink caffeinated beverages for at least 24 h prior to ECG and respiratory signals recording.

### Study Protocol

This was a prospective observational case–controlled study. On the day of HRV study, the morning doses of aminophylline, *β*2‐agonists, and steroid were requested to postpone until the completion of the study in the afternoon so that the drug effects would not be too strong to interfere with the result of HRV analysis. If the patient felt uncomfortable or dyspneic, then the study was discontinued and the medication was taken by the patient immediately.

### Physiological measurements

All subjects were studied in supine position in a quiet air‐conditioned room with constant temperature around 25°C and suitable humidity. After a 5‐min rest in supine position, a trend of lead II ECG signals and a trend of nostril airflow signals were picked up by a multichannel recorder (Biopac MP35, Biopac Systems, Inc., Goleta, CA), and transmitted to a notebook computer for recording for 15 min so that at least 512 RR intervals could be obtained. The sampling frequency of ECG and airflow signals recording was 500 Hz. During the period of recording, the patient was asked to close their eyes and relax on bed so that the interferences from the environment could be minimized. The analysis programs of cross spectral and traditional HRV analyses were programmed by using Mathcad 13 software (Mathsoft Inc., Cambridge, MA).

The airflow signals of the healthy subjects and COPD patients were collected by a hot‐wire thermister (SS6L temperature transducer, BIOPAC systems Inc., Goleta, CA) attached to the skin surface directly underneath the nostril of the subject at one end, and connected to the data acquisition system of the multichannel recorder at the other. The operating principles for the hot‐wire airflow measurement is thermal anemometry, which is the most common method used to measure instantaneous airflow velocity by measuring the total heat loss of a heating element and correlates the output signal to the flow rate of the fluid. When the air of breath flows through the sensor, it measures the total heat loss. The sensor converts pressure into an analog electrical voltage as output signal, which is proportional to airflow. The temperature at the nostrils is inversely proportional to the airflow in and out of the nostril. The recorded variations in temperature in the exhaled airflow during breathing can be used to indicate respiratory pattern.

After recording ECG and nostril airflow signals, the pulmonary function test (Brentwood Diagnostic Workstation, Cardiology Shop, Boston, MA) was performed. The forced expiratory volume in the first second/forced vital capacity (FEV_1_/FVC) and %FEV_1_ (% predicted of FEV_1_) were used as the indices of airflow obstruction.

### HRV analysis

The method of HRV analysis has been published previously (Task Force of the European Society of Cardiology, the North American Society of Pacing and Electrophysiology, 1996; Wang et al. 2015). In brief, the recorded ECG signals were retrieved to measure the consecutive RRI, which are the time intervals between successive pairs of QRS complexes, using the software for the detection of R waves. The ectopic beats were replaced by interpolated beats placed midway between two normal beats before and after the ectopic beats. If the percentage of interpolation was greater than 5%, the data of the subject were excluded from the study.

Both time‐ and frequency‐domain HRV measures of 512 RRI were obtained for comparison. The time‐domain measures including the mean (mRRI), standard deviation (SD_RR_), coefficient of variation (CV_RR _= SD_RR_/mRRI), and root mean squared successive differences (RMSSD) of RRI were calculated using standard formulae. The zero‐frequency component or direct current was excluded before the calculation of power spectral density (PSD). The area under the spectral peaks within the frequency ranges of 0.003–0.4 Hz, 0.003–0.04 Hz, 0.04–0.15 Hz, 0.15–0.4 Hz were defined as the total power (TP), very low‐frequency power (VLFP), low‐frequency power (LFP), and high‐frequency power (HFP), respectively. The normalized VLFP (nVLFP = VLFP/TP) was used as the index of vagal withdrawal, renin–angiotensin modulation and thermoregulation; the normalized HFP (nHFP = HFP/TP) as the index of vagal modulation; the normalized LFP (nLFP = LFP/TP) as the index of combined sympathetic and vagal modulation; and the low‐/high‐ frequency power ratio (LHR = LFP/HFP) as the index of sympathovagal balance (Pagani et al. 1986).

### Cross‐spectral analysis of ECG and nostril airflow signals

A segment of ECG signals that contains 512 RRI and a segment of airflow signals of equal length were used for cross‐spectral analysis. The cross‐spectral density (CSD) of time series *x* and *y* as a function of frequency *f*, CSD_*xy*_(*f*), is the Fourier transform of the cross‐correlation function (CCF) between series *x* and *y* (Cerutti et al. 1995).

$$CSD_{xy}\left(f\right)=FT\left[\theta_{xy}\left(\tau \right)\right]=\frac{1}{N\Delta t}X\left(f\right)Y^{*}\left(f\right),$$

where *FT* denotes Fourier transformation, *θ*_*xy*_(*τ*) = ∑_*k*_ *x(k)y*(*k* + *τ*), is the CCF of series *x* and *y, N* is the sample size, *∆t* is the sampling interval, *X*(*f*) and *Y*(*f*) are the Fourier transforms of *x* and *y*, respectively, and Y*(*f*) is the complex conjugate of *Y*(*f*). The range of summation in the calculation of CCF was limited to the range of the available overlapped data.

The area under the spectral peaks within the frequency ranges of 0.003–0.4 Hz, 0.003–0.04 Hz, 0.04–0.15 Hz, and 0.15–0.4 Hz in the cross spectrum of ECG and nostril airflow were defined as the cross‐spectral total power (TPcs), very low‐frequency power (VLFPcs), low‐frequency power (LFPcs), and high‐frequency power (HFPcs), respectively. The normalized VLFPcs (nVLFPcs = VLFPcs/TPcs), normalized HFPcs (nHFPcs = HFPcs/TPcs), normalized LFPcs (nLFPcs = LFPcs/TPcs), and the low‐/high‐frequency power ratio (LHRcs = LFPcs/HFPcs) were defined in similar ways as those in the traditional HRV. The “cs” stands for “cross spectral”.

To compare the effect of cross‐spectral analysis on the changes in spectral measures between normal subjects and COPD patients, the percentage changes in spectral measures in each subject were calculated using the following formula:

%Χ_ _= [(Χcs ‐Χ)/(Χ)]×100%,

where Xcs stands for cross‐spectral measure and X stands for HRV measure.

### Statistical analysis

Statistical analysis was performed on HRV measures and corresponding cross‐spectral measures (SigmaPlot 13.0, SPSS Inc., Chicago, IL). Since the absolute powers of HRV such as TP, VLFP, LFP, and HFP and the corresponding cross‐spectral measures have different units, they cannot be compared statistically. Only the normalized powers and power ratio of traditional HRV and cross‐spectral measures were compared.

Unpaired Student t‐test or Mann–Whitney rank sum test were employed to compare the normally distributed or distribution‐free clinical data, HRV measures, cross‐spectral measures, and the percentage changes in spectral measures between normal subjects and COPD patients. Wilcoxon signed‐rank test was used to compare the HRV measures and the corresponding cross‐spectral measures.

Linear regression analysis was employed to assess the relationships between HRV measures and cross‐spectral measures, and between the clinical characteristics and two kinds of measures, and between clinical characteristics and the percentage changes in spectral measures in normal subjects and COPD patients. A *P *<* *0.05 was considered statistically significant.

## Results

Twenty‐three healthy subjects and 23 COPD patients were included in the study. All data are presented as median and interquartile ranges (25th to 75th percentiles). Table 1 shows the baseline characteristics of the control subjects and COPD patients. Most clinical characteristics were not significantly different between the control subjects and COPD patients. However, the forced vital capacity (FVC), % predicted FVC (%FVC), forced expiratory volume in the first second (FEV_1_), % predicted FEV_1_ (%FEV_1_), and FEV_1_/FVC of the COPD patients were all significantly decreased, as compared with the control subjects.

**Table 1:** Comparisons of the baseline characteristics between control subjects and COPD patients

	Control (*n* = 23)	COPD (*n* = 23)	*P* value
Gender (M/F)	20/3	19/4	NS^a^
Age (year)	77 (76–78)	81 (76–83)	NS
Body height (cm)	163 (159–170)	162 (158–168)	NS
Body weight (cm)	64 (58–70)	64 (53–72)	NS
BMI (kg/m^2^)	23.8 (22.4–25.2)	24.1 (21.7–28.1)	NS
SBP (mmHg)	134 (126–148)	147 (123–151)	NS
DBP (mmHg)	69 (61–73)	72 (64–82)	NS
PP (mmHg)	65 (59–75)	65 (58–78)	NS
FVC(liter)	2.8 (2.5–3.4)	2.0 (1.6–2.4)	<0.001
%FVC(%)	100.0 (91.0–114.9)	66.9 (55.6–90.6)	<0.001
FEV_1_(liter)	2.3 (1.9–2.6)	1.0 (0.8–1.4)	<0.001
FVI_1_/FVC(%)	78.0 (73.0–81.0)	55.7 (46.0–62.5)	<0.001

Values are expressed as medians (IQR, 25–75%).

Mann–Whitney rank sum test. COPD, chronic obstructive pulmonary disease; BMI, body mass index; SBP, systolic blood pressure; DBP, diastolic blood pressure; PP, pulse pressure; FVC, forced vital capacity; %FVC, percentage of FVC to the expected FVC; FEV_1_, forced expiratory volume in the first second; %FEV_1_,; percentage of FEV_1_ to the expected FEV_1_; FEV_1_/FVC, ratio of FEV_1_ to FVC; NS, not significant.

a Chi‐square test.

**Figure 1:**
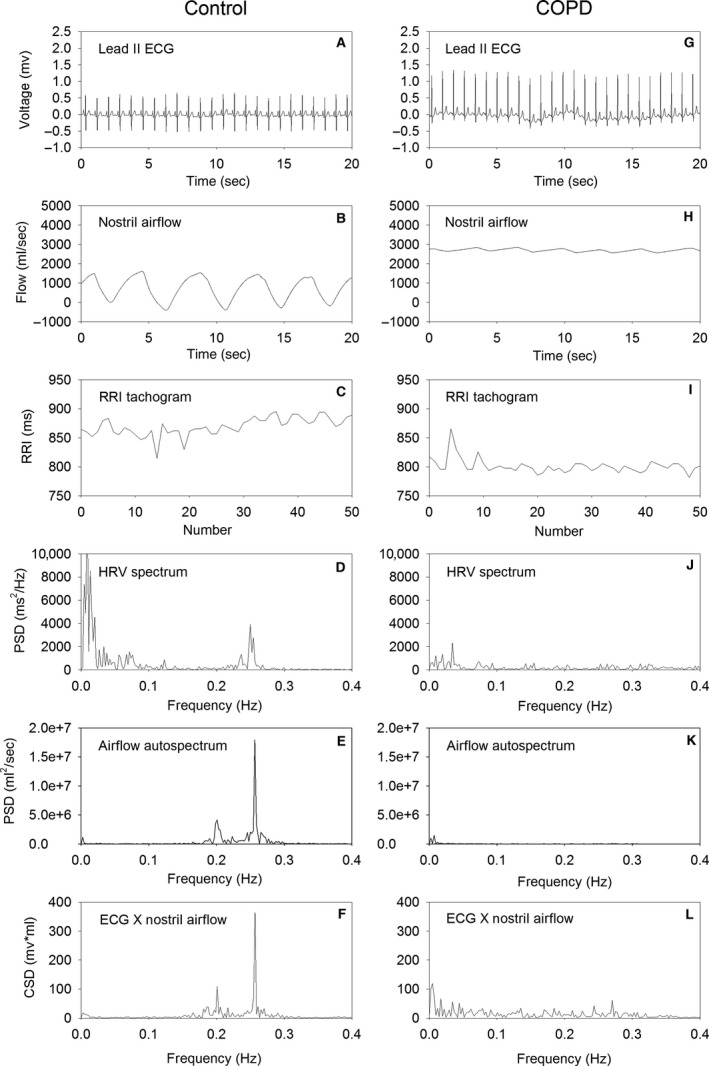
The ECG waveform, nostril airflow, RRI tachogram, heart rate variability spectrum, autospectrum of nostril airflow, and cross spectrum of ECG and nostril airflow in a representative healthy subject and a representative chronic obstructive pulmonary disease (COPD) patient. The ordinate (Y axis) of the panels of the representative COPD patient is scaled to the same extent as that of the normal subject so that the figures of the representative COPD patient and normal subject can be compared visually on the same footing.

Figure 1 shows the lead II ECG waveform, nostril airflow, RRI tachogram, HRV spectrum, airflow autospectrum, and the cross spectrum of ECG and nostril airflow signals in a representative normal subject and a representative COPD patient. The scale of the ordinate is set to be the same for both normal subjects and COPD patients so that the magnitude of waveform and spectral peaks can be compared visually. The nostril airflow of the COPD patients had smaller amplitude of fluctuation than that of normal subjects. In the HRV spectrum, airflow autospectrum, and cross spectrum of ECG and nostril airflow, the spectral peaks at the respiratory frequency in the COPD patients were smaller than those of normal subjects.

**Figure 2:**
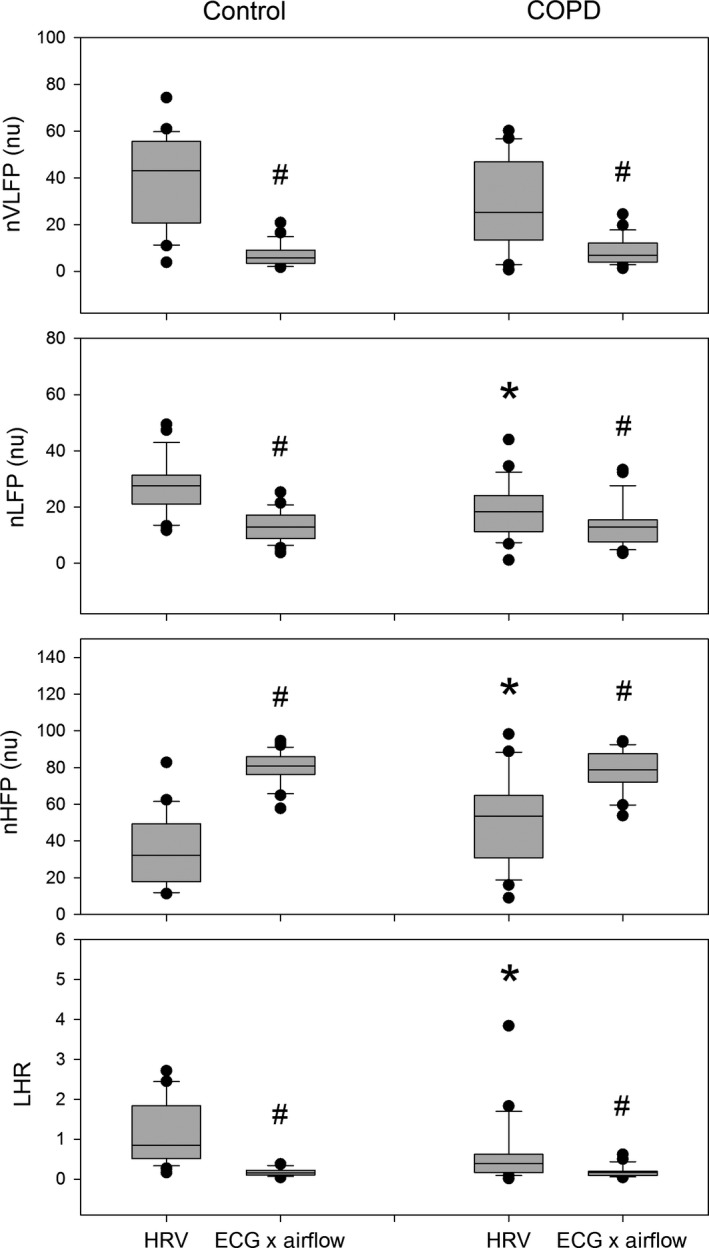
The comparison of normalized heart rate variability measures and normalized cross‐spectral measures of ECG and nostril airflow in normal subjects and chronic obstructive pulmonary disease patients, and between normal subjects and COPD patients. #*P* < 0.05 versus corresponding heart rate variability (HRV) measure in the same group. *P < 0.05 versus corresponding HRV measure in the control group.

Figure 2 shows that the TP, HFP, and nHFP of COPD patients were significantly greater than those of normal subjects. In addition, cross‐spectral analysis could lead to a significant increase in nHFPcs and significant decrease in nVLFPcs, nLFPcs, and LHRcs in both normal subjects and COPD patients, as compared with their corresponding HRV measures.

Table 2 shows that the time‐domain HRV measures such as SD_RR_, CV_RR_, and rMSSD of COPD patients were significantly greater than those of normal subjects. In the frequency‐domain, the TP, HFP, and nHFP of COPD patients were significantly greater, while the nLFP and LHR of COPD patients were significantly smaller, than those of normal subjects. However, all cross‐spectral measures of ECG and nostril airflow of COPD patients were not significantly different from those of normal subjects. Further analysis showed that the %nHFP of COPD patients was significantly smaller, while the %LHR of COPD patients was significantly greater, than those of normal subjects. That is, the magnitude of enhancement in nHFPcs and the magnitude of suppression in LHRcs in COPD patients were smaller than those of normal subjects.

**Table 2:** Comparisons of the time‐ and frequency‐domain HRV measures, cross‐spectral measures of ECG and nostril airflow, and the percentage changes in spectral measures between normal subjects and COPD patients

	Control (*n* = 23)	COPD (*n* = 23)	*P* value
Time‐domain HRV measures			
HR (bpm)	68.2 (61.1–76.7)	67.7 (64.2–75.0)	NS
mRRI (msec)	867 (801–985)	862 (794–939)	NS
SD_RR_ (msec)	23 (18–33)	38 (25–62)	0.004
CV_RR_ (%)	2.72 (1.72–3.76)	4.91 (3.09–6.72)	0.003
rMSSD (msec)	15.5 (11.5–39.1)	51.4 (23.2–95.8)	0.001
Frequency–domain HRV measures			
TP (msec^2^)	204.1 (127.6–422.8)	449.6 (195.6–1147.0)	0.015
VLFP (msec^2^)	70.4 (49.1–97.0)	73.6 (30.8–184.0)	NS
LFP (msec^2^)	52.1 (28.9–74.0)	65.8 (27.1–266.2)	NS
HFP (msec^2^)	41.1 (25.4–181.3)	223.7(53.3–795.3)	0.006
nVLFP (nu)	43.0 (20.8–55.7)	25.2 (13.3–46.9)	NS
nLFP (nu)	27.5 (21.1–31.4)	18.3 (11.3–24.2)	0.004
nHFP (nu)	32.2 (17.9–49.4)	53.6 (31.0–64.8)	0.007
LHR	0.85 (0.51–1.84)	0.39 (0.17–0.62)	<0.001
Cross–spectral measures			
TPcs (mv × mL/sec)	1.05 (0.58–2.25)	1.07 (0.84–1.88)	NS
VLFPcs (mv × mL/sec)	0.06 (0.04–0.10)	0.07 (0.04–0.17)	NS
LFPcs (mv × mL/sec)	0.12 (0.08–0.23)	0.13 (0.07–0.21)	NS
HFPcs (mv × mL/sec)	0.84 (0.46–1.54)	0.84 (0.65–1.61)	NS
nVLFPcs (nu)	5.7 (3.5–9.1)^a^	6.9 (4.0–12.2)^a^	NS
nLFPcs (nu)	12.9 (8.8–17.2)^a^	12.9 (7.5–15.5)^a^	NS
nHFPcs (nu)	80.8 (76.2–85.9)^a^	78.8 (72.2–87.5)^a^	NS
LHRcs	0.16 (0.10–0.22)^a^	0.16 (0.09–0.20)^a^	NS
Percentage changes in spectral measures			
%nVLFP (%)	−82.9 (–90.6 to −72.9)	−67.6 (−86.8 to−32.2)	NS
%nLFP (%)	−45.0 (−73.8 to −31.6)	−45.6 (−64.0–12.4)	NS
%nHFP (%)	141.5 (78.5 – 347.4)	47.0 (13.6–134.2)	0.008
%LHR (%)	−83.9 (−90.9 to −73.0)	−59.1 (−87.7 to −2.6)	0.041

Values are expressed as medians (IQR, 25–75%).

HRV, heart rate variability; ECG, electrocardiography; COPD, chronic obstructive pulmonary disease; HR, heart rate; SD_RR_, standard deviation of RR intervals; CV_RR_, coefficient of variation in RR intervals; TP, total power; VLFP, very low‐frequency power; LFP, low‐frequency power; HFP, high‐frequency power; nVLFP, normalized VLFP; nLFP, normalized LFP; nHFP; normalized HFP; LFP/HFP, low‐/high‐frequency power ratio; ms, millisecond; nu, normalized unit; mv, millivolt; ml, milliliter; cs, cross spectral; NS, not significant.

*P* < 0.05 versus corresponding HRV measure.

**Figure 3:**
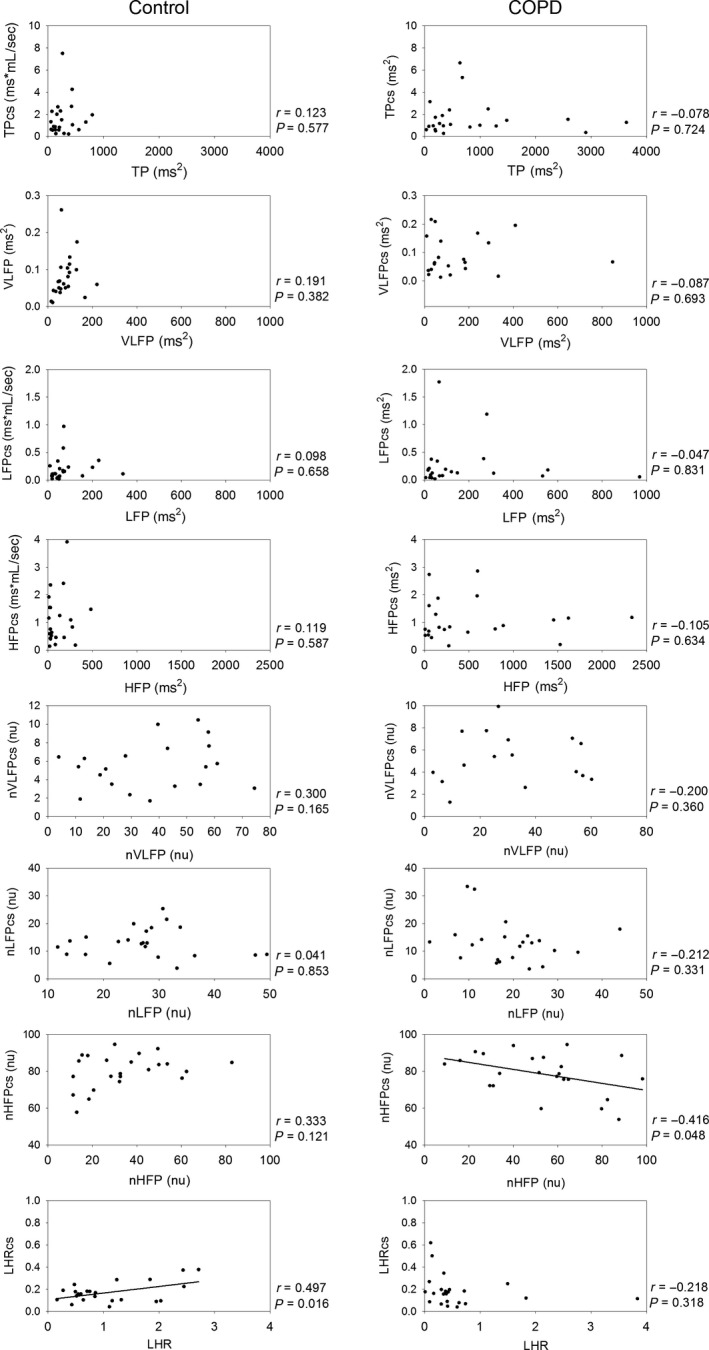
Linear correlations between heart rate variability measures and cross‐spectral measures of ECG and nostril airflow in normal subjects and chronic obstructive pulmonary disease patients.

Figure 3 shows that most cross‐spectral measures of ECG and nostril airflow did not correlate significantly with their HRV counterparts in both normal subjects and COPD patients. Only the LHRcs correlated significantly and positively with the LHR in normal subjects, and only the nHFPcs correlated significantly and negatively with the nHFP in COPD patients, suggesting that cross‐spectral measures of ECG and nostril airflow were related to, but not equal to, their corresponding HRV measures.

Table 3 shows that FEV_1_/FVC correlated significantly and positively with nLFP in normal subjects only. Other HRV measures, cross‐spectral measures or the percentage changes in spectral measures did not correlate with FEV_1_/FVC in either normal subjects or COPD patients. Table 4 shows that the FVC correlated significantly and positively with LHR in normal subjects only. Other HRV measures, cross‐spectral measures or the percentage changes in spectral measures did not correlate with FVC in either normal subjects or COPD patients.

**Table 3:** The correlations between FEV_1_/FVC and HRV measures, cross‐spectral measures of ECG and nostril airflow, and the percentage changes in spectral measures

	Control (*n* = 23)	COPD (*n* = 23)
HRV measures		
nVLFP (nu)	*r* = −0.009; *P* = 0.967	*r* = −0.074; *P* = 0.737
nLFP (nu)	*r* = 0.445; *P* = 0.034^a^	*r* = −0.117; *P* = 0.595
nHFP (nu)	*r* = −0.213; *P* = 0.329	*r* = 0.104; *P* = 0.637
LHR	*r* = 0.106; *P* = 0.631	*r* = −0.020; *P* = 0.927
Cross–spectral measures		
nVLFPcs (nu)	*r* = 0.197; *P* = 0.367	*r* = −0.054; *P* = 0.806
nLFPcs (nu)	*r* = 0.127; *P* = 0.562	*r* = 0.210; *P* = 0.336
nHFPcs (nu)	*r* = −0.177; *P* = 0.420	*r* = −0.116; *P* = 0.599
LHRcs	*r* = 0.125; *P* = 0.569	*r* = 0.202; *P* = 0.355
Percentage changes in spectral measures		
%nVLFP (%)	*r* = −0.201; *P* = 0.359	*r* = −0.187; *P* = 0.393
%nLFP (%)	*r* = −0.190; *P* = 0.385	*r* = −0.133; *P* = 0.544
%nHFP (%)	*r* = −0.023; *P* = 0.918	*r* = −0.021; *P* = 0.923
%LHR (%)	*r* = −0.283; *P* = 0.190	*r* = −0.111; *P* = 0.614

FEV_1_/FVC, ratio of forced expiratory volume in the first second to forced vital capacity; HRV, heart rate variability; ECG, electrocardiography; nVLFP, normalized very low‐frequency power; nLFP, normalized low‐frequency power; nHFP; normalized high‐frequency power; LFP/HFP, low‐/high‐frequency power ratio; COPD, chronic obstructive pulmonary disease; cs, cross spectral.

a *P* < 0.05.

**Table 4:** The correlations between FVC and HRV measures, cross‐spectral measures of ECG and nostril airflow, and the percentage changes in spectral measures

	Control (*n* = 23)	COPD (*n* = 23)
HRV measures		
nVLFP (nu)	*r* = 0.211; *P* = 0.335	*r* = 0.202; *P* = 0.355
nLFP (nu)	*r* = 0.221; *P* = 0.311	*r* = 0.368; *P* = 0.084
nHFP (nu)	*r* = −0.326; *P* = 0.129	*r* = −0.303; *P* = 0.160
LHR	*r* = 0.555; *P* = 0.006^a^	*r* = 0.203; *P* = 0.352
Cross‐spectral measures		
nVLFPcs (nu)	*r* = 0.105; *P* = 0.632	*r* = −0.054; *P* = 0.806
nLFPcs (nu)	*r* = 0.369; *P* = 0.083	*r* = 0.210; *P* = 0.336
nHFPcs (nu)	*r* = −0.271; *P* = 0.211	*r* = −0.116; *P* = 0.599
LHRcs	*r* = 0.380; *P* = 0.073	*r* = 0.202; *P* = 0.355
Percentage changes in spectral measures		
%nVLFP (%)	*r* = −0.171; *P* = 0.436	*r* = 0.057; *P* = 0.798
%nLFP (%)	*r* = −0.067; *P* = 0.762	*r* = 0.159; *P* = 0.469
%nHFP (%)	*r* = 0.351; *P* = 0.101	*r* = −0.138; *P* = 0.531
%LHR (%)	*r* = −0.213; *P* = 0.328	*r* = 0.070; *P* = 0.749

FVC, forced vital capacity; HRV, heart rate variability; ECG, electrocardiography; nVLFP, normalized very low‐frequency power; nLFP, normalized low‐frequency power; nHFP; normalized high‐frequency power; LFP/HFP, low‐/high‐frequency power ratio; COPD, chronic obstructive pulmonary disease; cs, cross spectral.

a *P* < 0.05.

Table 5 shows that a greater body mass index (BMI) was associated with a greater nVLFPcs, %nVLFP, %nLFP, and %LHR, and with a smaller nLFP and nHFPcs in normal subjects; whereas a greater BMI was associated with a greater nHFP and LHRcs, and with a smaller nVLFP, nHFPcs, and %nHFP in COPD patients. It is interesting to note that the BMI correlated significantly and positively with nHFP, while it correlated significantly and negatively with nHFPcs in COPD patients.

**Table 5:** The correlations between BMI and HRV measures, cross‐spectral measures of ECG and nostril airflow, and the percentage changes in spectral measures

	Control (*n* = 23)	COPD (*n* = 23)
HRV measures		
nVLFP (nu)	*r* = 0.066; *P* = 0.766	*r* = −0.534; *P* = 0.009[Link]
nLFP (nu)	*r* =−0.481; *P* = 0.020^a^	*r* = −0.261; *P* = 0.229
nHFP (nu)	*r* = 0.174; *P* = 0.428	*r* = 0.522; *P* = 0.011^a^
LHR	*r* = −0.118; *P* = 0.592	*r* = −0.396; *P* = 0.061
Cross‐spectral measures		
nVLFPcs (nu)	*r* = 0.477; *P* = 0.021^a^	*r* = 0.380; *P* = 0.073
nLFPcs (nu)	*r* = 0.397; *P* = 0.061	*r* = 0.375; *P* = 0.078
nHFPcs (nu)	*r* = −0.479; *P* = 0.021^a^	*r* = −0.453; *P* = 0.030^a^
LHRcs	*r* = 0.394; *P* = 0.063	*r* = 0.448; *P* = 0.032^a^
Percentage changes in spectral measures		
%nVLFP (%)	*r* = 0.436; *P* = 0.038^a^	*r* = 0.059; *P* = 0.789
%nLFP (%)	*r* = 0.489; *P* = 0.018^a^	*r* = 0.046; *P* = 0.835
%nHFP (%)	*r* = −0.101; *P* = 0.645	*r* = −0.494; *P* = 0.017^a^
%LHR (%)	*r* = 0.513; *P* = 0.012^a^	*r* = 0.095; *P* = 0.667

BMI, body mass index; HRV, heart rate variability; ECG, electrocardiography; nVLFP, normalized very low‐frequency power; nLFP, normalized low‐frequency power; nHFP; normalized high‐frequency power; LFP/HFP, low‐/high‐frequency power ratio; COPD, chronic obstructive pulmonary disease; cs, cross spectral.

a *P* < 0.05.

## Discussion

Since respiration is an important ingredient of HRV, we investigated the cross‐spectral measures of ECG and nostril airflow signals and compared them with their corresponding HRV measures in both normal controls and COPD patients. We found that cross‐spectral analysis of ECG and nostril airflow signals could significantly intensify the high frequency or respiratory component and suppress other nonrespiratory components in the cross spectrum to such extent that all cross‐spectral measures of ECG and nostril airflow of COPD patients were not significantly different from those of normal subjects.

The %nHFP and %LHR in either normal subjects or COPD patients were related to each other because LHR is equal to nLFP divided by nHFP. Thus, the increase in nHFPcs and the decrease in LHRcs as compared with their counterparts in HRV (Fig. 2 and Table 2) could be understood to be caused mainly by the increase in the high‐frequency component due to cross‐spectral analysis. In COPD patients, though the nHFP of conventional HRV was significantly greater and the nLFP and LHR were significantly smaller, the %nHFP was significantly smaller, and the %LHR was significantly greater, than those of normal subjects. It seems that the high‐frequency component in the ECG tracing was already enhanced in the COPD patients so that the room for further enhancement in high‐frequency component by cross‐spectral analysis with the help of nostril airflow signals was limited. Cross‐spectral analysis allows one to find the common features of two signals, and disclose the dependence of one signal over the other (Rangayyan 2002). Therefore, the cross‐spectral measures of ECG and nostril airflow signals should contain information related to not only respiration but also cardiovascular‐related systems. The finding of significant correlations between BMI and the percentage changes in spectral measures in both normal subjects and COPD patients seemed to confirm this speculation, because BMI is an important factor in assessing cardiovascular morbidity and mortality (Aronis et al. 2015; Guwatudde et al. 2015; Owen et al. 2015). For instance, the BMI, abdominal circumference, and total fat mass are associated with the risk of atrial fibrillation among white and black older adults (Aronis et al. 2015). A higher BMI at 21 years of age is associated with later diabetes incidence but not myocardial infarction or stroke, while higher BMI in middle age is strongly associated with all outcomes (Owen et al. 2015). Therefore, the significant negative correlation between BMI and nHFPcs and %nHFP in COPD patients suggested that cross‐spectral analysis of ECG and nostril airflow might be used to evaluate the cardiovascular morbidity and mortality in COPD patients in the future. Further studies are needed to elucidate the clinical significance of the cross‐spectral measures and the percentage changes in spectral measures in COPD patients.

Our findings about the relation between cross‐spectral measures of ECG and nostril airflow and the cardiovascular status of the patients are rather premature at the present stage. This is inevitable because this study is the first one using cross‐spectral analysis to find the common characteristics of ECG and respiration and to compare the cross‐spectral measures of ECG and nostril airflow with their corresponding HRV measures in normal control and COPD patients. With more studies using this technique to investigate the cardiopulmonary interaction in various kinds of physiological and pathological conditions, the clinical meaning and significance of cross‐spectral measures of ECG and nostril airflow will become clearer in the future.

Respiratory movements are an integral part of HRV. However, the influence of respiratory movements on ECG might not be apparent without the use of appropriate technique. The current HRV analysis is one of the techniques that can display the influence of respiratory movements on ECG. Cross‐spectral analysis with the help of respiratory signals might be another potential technique. Different techniques should uncover different aspects of the cardiopulmonary interaction in the patients. The differences between cross‐spectral measures of ECG and nostril airflow and their corresponding HRV measures in this study showed that cross‐spectral analysis of ECG and nostril airflow is really not the same as traditional HRV analysis. The positive correlation between BMI and nHFP and the negative correlation between BMI and nHFPcs in COPD patients (Table 5) showed that cross‐spectral analysis of ECG and nostril airflow might give us new information about the cardiopulmonary interaction in patients with COPD and possibly other kinds of diseases.

Since the patients included in this study were clinically stable patients, the postpone in taking one dose of medication should not cause dyspneic episode in them. The reason why we requested the patients to skip one dose of medication was to avoid the acute drug effects so that the effects of the drugs would not be too strong to interfere with the result of HRV analysis. If the patient felt uncomfortable or dyspneic, then the study was discontinued and the medication was given to the patient immediately. No patient encountered this situation in this study.

In conclusion, cross‐spectral analysis of ECG and respiratory signals could lead to a reduced enhancement in the high‐frequency component in the cross spectrum of ECG and nostril airflow in COPD patients. The magnitude of reduced enhancement in the high‐frequency component in the cross spectrum of ECG and nostril airflow was related to the BMI of the COPD patients. The cross‐spectral analysis of ECG and nostril airflow might be used to assess the cardiovascular‐related functions of COPD patients in the future.

## Conflict of Interest

No conflicts of interest, financial, or otherwise, are declared by the author(s).

## References

[phy212763-bib-0001] Akselrod, S. , D. Gordon , F. A. Ubel , D. C. Shannon , A. C. Berger , and R. J. Cohen . 1981 Power spectrum analysis of heart rate fluctuation: a quantitative probe of beat‐to‐beat cardiovascular control. Science 213:220–222. 6166045 10.1126/science.6166045

[phy212763-bib-0002] American Thoracic Society . 1987 Standards for the diagnosis and care of patients with chronic obstructive pulmonary disease and asthma. Am. Rev. Respir. Dis. 136:225–244. 3605835 10.1164/ajrccm/136.1.225

[phy212763-bib-0003] Aronis, K. N. , N. Wang , C. L. Phillips , E. J. Benjamin , G. M. Marcus , A. B. Newman , et al. 2015 HealthABC study. Associations of obesity and body fat distribution with incident atrial fibrillation in the biracial health aging and body composition cohort of older adults. Am. Heart J. 170:498–505. 26385033 10.1016/j.ahj.2015.06.007 PMC4575766

[phy212763-bib-0004] Brown, T. E. , L. A. Beightol , J. Koh , and D. L. Eckberg . 1993 Important influence of respiration on human R‐R interval power spectra is largely ignored. J. Appl. Physiol. 75:2310–2317. 8307890 10.1152/jappl.1993.75.5.2310

[phy212763-bib-0005] Cerutti, S. , A. M. Bianchi , and L. T. Mainardi . 1995 Spectral analysis of the heart rate variability signalsPp:63–74 *in*: Malik M., Cam A. J., eds. Herat Rate Variability. Futura Publishing Company, Inc, New York.

[phy212763-bib-0006] Chen, W. L. , G. Y. Chen , and C. D. Kuo . 2006 Hypoxemia and autonomic nervous dysfunction in patients with chronic obstructive pulmonary disease. Respir. Med. 100:1547–1553. 16488587 10.1016/j.rmed.2006.01.006

[phy212763-bib-0007] Guwatudde, D. , G. Mutungi , R. Wesonga , R. Kajjura , H. Kasule , J. Muwonge , et al. 2015 The epidemiology of hypertension in Uganda: findings from the national non‐communicable diseases risk factor survey. PLoS ONE 10:e0138991. 26406462 10.1371/journal.pone.0138991 PMC4583385

[phy212763-bib-0008] Hirsch, J. A. , and B. Bishop . 1981 Respiratory sinus arrhythmia in humans: how breathing pattern modulates heart rate. Am. J. Physiol. Heart Circ. Physiol. 241:H620–H629. 10.1152/ajpheart.1981.241.4.H620 7315987

[phy212763-bib-0009] Kay, S. M. , and S. L. Marple Jr **.** 1981 Spectrum analysis: a modem perspective. Proc. IEEE 69:1380–1419.

[phy212763-bib-0010] Kleiger, R. E. , P. K. Stein , and J. T. Bigger . 2005 Heart rate variability: measurement and clinical utility. Ann. Noninvasive Electrocardiol. 10:88–101. 15649244 10.1111/j.1542-474X.2005.10101.x PMC6932537

[phy212763-bib-0011] Owen, C. G. , V. V. Kapetanakis , A. R. Rudnicka , A. K. Wathern , L. Lennon , O. Papacosta , et al. 2015 Body mass index in early and middle adult life: prospective associations with myocardial infarction, stroke and diabetes over a 30‐year period: the British regional heart study. BMJ Open 5:e008105. 10.1136/bmjopen-2015-008105 PMC4577944 26373398

[phy212763-bib-0012] Pagani, M. , F. Lombardi , S. Guzzetti , O. Rimoldi , R. Furlan , P. Pizzinelli , et al. 1986 Power spectral analysis of heart rate and arterial pressure variabilities as a marker of sympatho‐vagal interaction in man and conscious dog. Circ. Res. 59:178–193. 2874900 10.1161/01.res.59.2.178

[phy212763-bib-0013] Rangayyan, R. M. 2002 Biospectral signal analysis: a case‐study approach. IEEE Press, Wiley‐Interscience, John Wiley & Sons Inc, New York.

[phy212763-bib-0014] Task Force of the European Society of Cardiology, the North American Society of Pacing and Electrophysiology . 1996 Heart rate variability: standards of measurement, physiological interpretation, and clinical use. Circulation 93:1043–1065. 8598068

[phy212763-bib-0015] Volterrani, M. , S. Scalvini , G. Mazzuero , P. Lanfranchi , R. Colombo , A. L. Clark , et al. 1994 Decreased heart rate variability in patients with chronic obstructive pulmonary disease. Chest 106:1432–1437. 7956396 10.1378/chest.106.5.1432

[phy212763-bib-0016] Wang, C. H. , A. M. L. Fan , C. Lin , and C. D. Kuo . 2015 Low‐altitude mountain tourism increases overall heart rate variability and decreases heart rate and blood pressures in healthy adults. J. Clin. Exp. Cardiol. 6:357–366.

